# *N*′-(3-Hy­droxy­benzyl­idene)-4-methyl­benzohydrazide

**DOI:** 10.1107/S1600536812021897

**Published:** 2012-05-19

**Authors:** Ji-Lai Liu, Ming-Hui Sun, Jing-Jun Ma

**Affiliations:** a Hebei Changshan Biochemical Pharmaceutical Co. Ltd, Shijiazhuang Hebei 050800, People’s Republic of China; b Department of Economics and Management, Hebei North University, Zhangjiakou Hebei 075000, People’s Republic of China; c Hebei Key Laboratory of Bioinorganic Chemistry, College of Sciences, Agricultural University of Hebei, Baoding 071001, People’s Republic of China

## Abstract

The title compound, C_15_H_14_N_2_O_2_, was obtained from the reaction of 3-hy­droxy­benzaldhyde and 4-methyl­benzo­hydrazide in methanol. In the mol­ecule, the benzene rings form a dihedral angle of 2.9 (3)°. In the crystal, N—H⋯O and O—H⋯O hydrogen bonds link the mol­ecules into layers parallel to (101). The crystal packing also exhibits π–π inter­actions between the aromatic rings [centroid–centroid distance = 3.686 (4) Å].

## Related literature

For the biological activity of benzohydrazide compounds, see: El-Sayed *et al.* (2011 ▶); Horiuchi *et al.* (2009 ▶). For benzohydrazide coordination compounds, see: El-Dissouky *et al.* (2010 ▶); Zhang *et al.* (2010 ▶). For standard bond lengths, see: Allen *et al.* (1987 ▶). For the crystal structures of similar compounds, see: Suleiman Gwaram *et al.* (2010 ▶); Liu *et al.* (2011 ▶); Zhang *et al.* (2012 ▶).
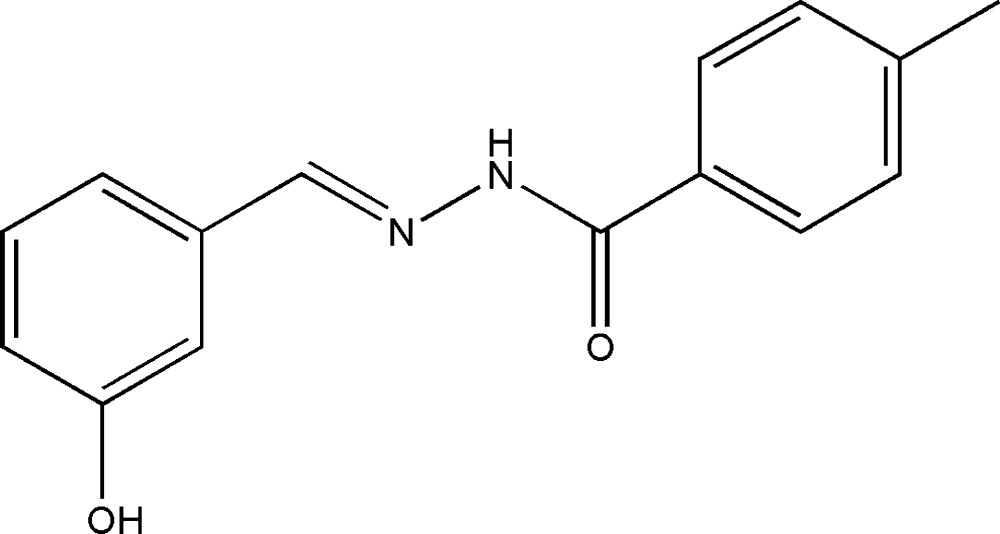


## Experimental

### 

#### Crystal data

C_15_H_14_N_2_O_2_
*M* *_r_* = 254.28
Monoclinic, 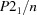

*a* = 11.5203 (14) Å
*b* = 8.7228 (12) Å
*c* = 13.5793 (19) Å
β = 106.889 (2)°
*V* = 1305.7 (3) Å^3^
*Z* = 4
Mo *K*α radiation
μ = 0.09 mm^−1^
*T* = 298 K
0.17 × 0.15 × 0.15 mm

#### Data collection

Bruker SMART 1K CCD area-detector diffractometer
Absorption correction: multi-scan (*SADABS*; Sheldrick, 1996 ▶) *T*_min_ = 0.985, *T*_max_ = 0.987
5767 measured reflections
2193 independent reflections
1013 reflections with *I* > 2σ(*I*)
*R*_int_ = 0.070

#### Refinement

*R*[*F* ^2^ > 2σ(*F* ^2^)] = 0.057
*wR*(*F* ^2^) = 0.160
*S* = 0.96
2193 reflections
176 parameters
1 restraint
H atoms treated by a mixture of independent and constrained refinement
Δρ_max_ = 0.19 e Å^−3^
Δρ_min_ = −0.19 e Å^−3^

### 

Data collection: *SMART* (Bruker, 2007 ▶); cell refinement: *SAINT* (Bruker, 2007 ▶); data reduction: *SAINT*; program(s) used to solve structure: *SHELXS97* (Sheldrick, 2008 ▶); program(s) used to refine structure: *SHELXL97* (Sheldrick, 2008 ▶); molecular graphics: *SHELXTL* (Sheldrick, 2008 ▶); software used to prepare material for publication: *SHELXTL*.

## Supplementary Material

Crystal structure: contains datablock(s) I, global. DOI: 10.1107/S1600536812021897/cv5303sup1.cif

Structure factors: contains datablock(s) I. DOI: 10.1107/S1600536812021897/cv5303Isup2.hkl

Supplementary material file. DOI: 10.1107/S1600536812021897/cv5303Isup3.cml

Additional supplementary materials:  crystallographic information; 3D view; checkCIF report

## Figures and Tables

**Table 1:** Hydrogen-bond geometry (Å, °)

*D*—H⋯*A*	*D*—H	H⋯*A*	*D*⋯*A*	*D*—H⋯*A*
N2—H2⋯O2^i^	0.90 (1)	2.10 (2)	2.926 (4)	152 (3)
O2—H2*B*⋯O1^ii^	0.82	1.91	2.713 (3)	166

Symmetry codes: (i) 
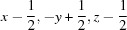
; (ii) 
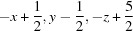
.

**Fig. 1.**
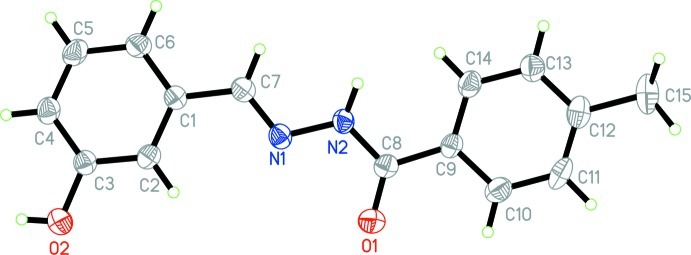
The molecular structure of (I), with the numbering scheme and displacement ellipsoids drawn at the 30% probability level.

## supplementary crystallographic information

### Comment

Benzohydrazide compounds are well known for their biological activities (El-Sayed *et al.*, 2011; Horiuchi *et al.*, 2009). In addition, benzohydrazide compounds have also been used as versatile ligands in coordination chemistry (El-Dissouky *et al.*, 2010, Zhang *et al.*, 2010). As a contribution to a structural study on hydrazone compounds, we present here the crystal structure of the title compound (I) obtained in the reaction of 3-hydroxybenzaldehyde with 4-methylbenzohydrazide in methanol.

In (I) (Fig. 1), two benzene rings form a dihedral angle of 2.9 (3)°. The bond lengths and angles are within normal ranges (Allen *et al.*, 1987), and agree well with those reported for related compounds (Suleiman Gwaram *et al.*, 2010; Liu *et al.*, 2011; Zhang *et al.*, 2012). Intermolecular N—H···O and O—H···O hydrogen bonds (Table 1) link the molecules into layers parallel to (101). The crystal packing exhibits π–π interactions between the aromatic rings [centroid-centroid distance = 3.686 (4) Å].

### Experimental

To a methanol solution (20 ml) of 3-hydroxybenzaldehyde (0.1 mmol, 12.2 mg) and 4-methylbenzohydrazide (0.1 mmol, 15.0 mg), a few drops of acetic acid were added. The mixture was refluxed for 1 h and then cooled to room temperature. The white crystalline solid was collected by filtration, washed with cold methanol and dried in air. Single crystals, suitable for X-ray diffraction, were obtained by slow evaporation of a methanol solution of the product in air.

### Refinement

The amino H-atom was located in a difference Fourier map and was refined with a distance restraint, N—H = 0.90 (1) Å. The hydroxy and C-bound H atoms were positioned geometrically (O—H 0.82 Å; C—H = 0.93 - 0.96 Å), and refined using a riding model, with *U*_iso_(H) = 1.2–1.5 *U*_eq_(C, O).

### Crystal data

**Table:**

C_15_H_14_N_2_O_2_	*F*(000) = 536
*M**_r_* = 254.28	*D*_x_ = 1.294 Mg m^−^^3^
Monoclinic, *P*2_1_/*n*	Mo *K*α radiation, λ = 0.71073 Å
*a* = 11.5203 (14) Å	Cell parameters from 1383 reflections
*b* = 8.7228 (12) Å	θ = 2.7–27.8°
*c* = 13.5793 (19) Å	µ = 0.09 mm^−^^1^
β = 106.889 (2)°	*T* = 298 K
*V* = 1305.7 (3) Å^3^	Prism, colourless
*Z* = 4	0.17 × 0.15 × 0.15 mm

### Data collection

**Table:**

Bruker SMART 1K CCD area-detector diffractometer	2193 independent reflections
Radiation source: fine-focus sealed tube	1013 reflections with *I* > 2σ(*I*)
Graphite monochromator	*R*_int_ = 0.070
ω scan	θ_max_ = 25.1°, θ_min_ = 2.8°
Absorption correction: multi-scan (*SADABS*; Sheldrick, 1996)	*h* = −10→13
*T*_min_ = 0.985, *T*_max_ = 0.987	*k* = −10→10
5767 measured reflections	*l* = −16→11

### Refinement

**Table:**

Refinement on *F*^2^	Primary atom site location: structure-invariant direct methods
Least-squares matrix: full	Secondary atom site location: difference Fourier map
*R*[*F*^2^ > 2σ(*F*^2^)] = 0.057	Hydrogen site location: inferred from neighbouring sites
*wR*(*F*^2^) = 0.160	H atoms treated by a mixture of independent and constrained refinement
*S* = 0.96	*w* = 1/[σ^2^(*F*_o_^2^) + (0.0672*P*)^2^] where *P* = (*F*_o_^2^ + 2*F*_c_^2^)/3
2193 reflections	(Δ/σ)_max_ < 0.001
176 parameters	Δρ_max_ = 0.19 e Å^−^^3^
1 restraint	Δρ_min_ = −0.19 e Å^−^^3^

### Special details

**Table:**

Geometry. All e.s.d.'s (except the e.s.d. in the dihedral angle between two l.s. planes) are estimated using the full covariance matrix. The cell e.s.d.'s are taken into account individually in the estimation of e.s.d.'s in distances, angles and torsion angles; correlations between e.s.d.'s in cell parameters are only used when they are defined by crystal symmetry. An approximate (isotropic) treatment of cell e.s.d.'s is used for estimating e.s.d.'s involving l.s. planes.
Refinement. Refinement of *F*^2^ against ALL reflections. The weighted *R*-factor *wR* and goodness of fit *S* are based on *F*^2^, conventional *R*-factors *R* are based on *F*, with *F* set to zero for negative *F*^2^. The threshold expression of *F*^2^ > σ(*F*^2^) is used only for calculating *R*-factors(gt) *etc*. and is not relevant to the choice of reflections for refinement. *R*-factors based on *F*^2^ are statistically about twice as large as those based on *F*, and *R*- factors based on ALL data will be even larger.

### Fractional atomic coordinates and isotropic or equivalent isotropic displacement parameters (Å^2^)

**Table:**

	*x*	*y*	*z*	*U*_iso_*/*U*_eq_	
N1	0.0696 (3)	0.2934 (3)	1.0157 (2)	0.0449 (8)	
N2	0.0285 (3)	0.3619 (3)	0.9196 (2)	0.0443 (8)	
O1	0.2096 (3)	0.4735 (3)	0.93581 (19)	0.0626 (8)	
O2	0.2664 (2)	0.0955 (3)	1.37553 (19)	0.0625 (8)	
H2B	0.2708	0.0441	1.4271	0.094*	
C1	0.0236 (3)	0.1217 (4)	1.1365 (3)	0.0392 (9)	
C2	0.1324 (3)	0.1476 (4)	1.2118 (3)	0.0434 (10)	
H2A	0.1869	0.2198	1.2012	0.052*	
C3	0.1591 (4)	0.0654 (4)	1.3023 (3)	0.0437 (10)	
C4	0.0790 (4)	−0.0422 (4)	1.3187 (3)	0.0509 (10)	
H4	0.0979	−0.0978	1.3797	0.061*	
C5	−0.0290 (4)	−0.0666 (4)	1.2441 (3)	0.0565 (11)	
H5	−0.0833	−0.1390	1.2548	0.068*	
C6	−0.0575 (3)	0.0154 (4)	1.1533 (3)	0.0506 (10)	
H6	−0.1312	−0.0009	1.1034	0.061*	
C7	−0.0067 (3)	0.2051 (4)	1.0389 (3)	0.0451 (10)	
H7	−0.0835	0.1934	0.9927	0.054*	
C8	0.1044 (4)	0.4477 (4)	0.8839 (3)	0.0427 (10)	
C9	0.0544 (4)	0.5105 (3)	0.7790 (3)	0.0410 (9)	
C10	0.1221 (4)	0.6163 (4)	0.7431 (3)	0.0578 (11)	
H10	0.1989	0.6433	0.7844	0.069*	
C11	0.0779 (5)	0.6825 (4)	0.6472 (3)	0.0640 (12)	
H11	0.1259	0.7524	0.6251	0.077*	
C12	−0.0351 (4)	0.6480 (4)	0.5838 (3)	0.0557 (11)	
C13	−0.1021 (4)	0.5414 (4)	0.6183 (3)	0.0566 (11)	
H13	−0.1784	0.5141	0.5762	0.068*	
C14	−0.0587 (4)	0.4735 (4)	0.7144 (3)	0.0520 (11)	
H14	−0.1064	0.4021	0.7356	0.062*	
C15	−0.0846 (4)	0.7240 (5)	0.4805 (3)	0.0806 (14)	
H15A	−0.1681	0.7508	0.4704	0.121*	
H15B	−0.0787	0.6547	0.4273	0.121*	
H15C	−0.0387	0.8150	0.4780	0.121*	
H2	−0.0506 (13)	0.367 (4)	0.884 (3)	0.080*	

### Atomic displacement parameters (Å^2^)

**Table:**

	*U*^11^	*U*^22^	*U*^33^	*U*^12^	*U*^13^	*U*^23^
N1	0.052 (2)	0.0353 (16)	0.0438 (19)	0.0031 (16)	0.0086 (17)	0.0024 (14)
N2	0.050 (2)	0.0366 (16)	0.0432 (19)	0.0005 (17)	0.0079 (17)	0.0067 (15)
O1	0.059 (2)	0.0708 (19)	0.0540 (17)	−0.0164 (15)	0.0102 (16)	−0.0015 (14)
O2	0.057 (2)	0.0703 (19)	0.0532 (17)	−0.0046 (15)	0.0047 (16)	0.0127 (14)
C1	0.045 (3)	0.0292 (18)	0.042 (2)	0.0046 (17)	0.010 (2)	−0.0003 (17)
C2	0.051 (3)	0.0307 (19)	0.051 (2)	−0.0002 (17)	0.018 (2)	−0.0006 (18)
C3	0.048 (3)	0.038 (2)	0.044 (2)	0.0030 (19)	0.012 (2)	0.0028 (18)
C4	0.063 (3)	0.038 (2)	0.051 (2)	0.000 (2)	0.017 (2)	0.0076 (19)
C5	0.071 (3)	0.039 (2)	0.062 (3)	−0.015 (2)	0.023 (3)	0.002 (2)
C6	0.054 (3)	0.038 (2)	0.055 (3)	−0.0064 (19)	0.008 (2)	−0.0009 (19)
C7	0.051 (3)	0.0312 (19)	0.049 (2)	0.0020 (19)	0.007 (2)	−0.0005 (18)
C8	0.052 (3)	0.0309 (19)	0.046 (2)	−0.0014 (19)	0.015 (2)	−0.0078 (18)
C9	0.053 (3)	0.0257 (18)	0.046 (2)	−0.0005 (18)	0.018 (2)	−0.0020 (17)
C10	0.062 (3)	0.052 (2)	0.061 (3)	−0.011 (2)	0.021 (2)	−0.003 (2)
C11	0.091 (4)	0.046 (2)	0.068 (3)	−0.011 (2)	0.043 (3)	0.010 (2)
C12	0.076 (3)	0.038 (2)	0.061 (3)	0.018 (2)	0.033 (3)	0.005 (2)
C13	0.062 (3)	0.052 (2)	0.054 (3)	0.002 (2)	0.015 (2)	0.005 (2)
C14	0.063 (3)	0.040 (2)	0.054 (3)	−0.003 (2)	0.018 (2)	0.0041 (19)
C15	0.114 (4)	0.066 (3)	0.068 (3)	0.029 (3)	0.037 (3)	0.026 (2)

### Geometric parameters (Å, º)

**Table:**

N1—C7	1.276 (4)	C6—H6	0.9300
N1—N2	1.388 (4)	C7—H7	0.9300
N2—C8	1.344 (4)	C8—C9	1.478 (5)
N2—H2	0.898 (10)	C9—C14	1.381 (4)
O1—C8	1.233 (4)	C9—C10	1.385 (5)
O2—C3	1.368 (4)	C10—C11	1.379 (5)
O2—H2B	0.8200	C10—H10	0.9300
C1—C6	1.380 (4)	C11—C12	1.369 (5)
C1—C2	1.386 (4)	C11—H11	0.9300
C1—C7	1.463 (4)	C12—C13	1.374 (5)
C2—C3	1.378 (4)	C12—C15	1.506 (5)
C2—H2A	0.9300	C13—C14	1.388 (4)
C3—C4	1.379 (4)	C13—H13	0.9300
C4—C5	1.374 (5)	C14—H14	0.9300
C4—H4	0.9300	C15—H15A	0.9600
C5—C6	1.381 (5)	C15—H15B	0.9600
C5—H5	0.9300	C15—H15C	0.9600
			
C7—N1—N2	114.9 (3)	O1—C8—C9	121.8 (4)
C8—N2—N1	119.9 (3)	N2—C8—C9	116.2 (4)
C8—N2—H2	117 (3)	C14—C9—C10	117.1 (3)
N1—N2—H2	122 (3)	C14—C9—C8	123.8 (3)
C3—O2—H2B	109.5	C10—C9—C8	119.0 (4)
C6—C1—C2	119.9 (3)	C11—C10—C9	121.3 (4)
C6—C1—C7	119.2 (4)	C11—C10—H10	119.3
C2—C1—C7	120.9 (3)	C9—C10—H10	119.3
C3—C2—C1	119.5 (3)	C12—C11—C10	121.6 (4)
C3—C2—H2A	120.2	C12—C11—H11	119.2
C1—C2—H2A	120.2	C10—C11—H11	119.2
O2—C3—C2	118.0 (3)	C11—C12—C13	117.4 (4)
O2—C3—C4	121.3 (3)	C11—C12—C15	121.5 (4)
C2—C3—C4	120.7 (4)	C13—C12—C15	121.1 (4)
C5—C4—C3	119.5 (4)	C12—C13—C14	121.6 (4)
C5—C4—H4	120.3	C12—C13—H13	119.2
C3—C4—H4	120.3	C14—C13—H13	119.2
C4—C5—C6	120.5 (4)	C9—C14—C13	120.9 (4)
C4—C5—H5	119.7	C9—C14—H14	119.6
C6—C5—H5	119.7	C13—C14—H14	119.6
C1—C6—C5	119.9 (4)	C12—C15—H15A	109.5
C1—C6—H6	120.1	C12—C15—H15B	109.5
C5—C6—H6	120.1	H15A—C15—H15B	109.5
N1—C7—C1	121.6 (4)	C12—C15—H15C	109.5
N1—C7—H7	119.2	H15A—C15—H15C	109.5
C1—C7—H7	119.2	H15B—C15—H15C	109.5
O1—C8—N2	122.0 (3)		

### Hydrogen-bond geometry (Å, º)

**Table:**

*D*—H···*A*	*D*—H	H···*A*	*D*···*A*	*D*—H···*A*
N2—H2···O2^i^	0.90 (1)	2.10 (2)	2.926 (4)	152 (3)
O2—H2*B*···O1^ii^	0.82	1.91	2.713 (3)	166

Symmetry codes: (i) *x*−1/2, −*y*+1/2, *z*−1/2; (ii) −*x*+1/2, *y*−1/2, −*z*+5/2.
